# Hospital and Other News

**Published:** 1922-12

**Authors:** 


### HOSPITAL AND OTHER NEWS.

Viscount Lascelles, K.G., is presiding, as chairman, over the Voluntary Hospitals Committee of the East and West Ridings of Yorkshire on November 28.

Six members of the nursing and six of the domestic staff of the Leeds Road Isolation Hospital have written to the Yorkshire press protesting against certain alleged grievances under which the patients and staff were said to suffer. The nursing staff's only "grievance," write the nurses, is "the use of margarine instead of butter. Otherwise there is very little cause for complaint."

The keeping of fowls by the members of the staff has been forbidden by the House Committee of the Cork District Mental Hospital, because, kept for profit and sold to attendants at current prices, they were sometimes housed in improper places, even in one of the cellars. This "unsanitary proceeding," and the roaming of hens about the grounds, were the decisive objections, which a committeeman is trying to have rescinded, because on two officers the prohibition has not been enforced.

The appeal issued last May on behalf of the Manchester hospitals was so unproductive that the Hospital Saturday Fund, and the institutions co-operating with it, have decided to make a second. While the trade depression in Manchester accounts for much, Manchester has long lagged behind cities of equal size in the sums that it raises for hospitals.

The tablet placed in the Middlesex Hospital Medical School to the memory of past and actual students who fell in the war has been unveiled by Lt.-Gen. Sir John Goodwin (Director-General, Army Medical Service) and dedicated by the Bishop of London. The list of names includes that of Capt. Fox Russell, V.C., M.C., whose portrait, by Mr. Ronald Gray, also hangs on the walls.

After an interval of 37 years a Bishop of Lincoln has once more preached at the anniversary service of the Nottingham General Hospital. This year for the first time the office of president is held by a lawyer. Mr. Frederick Acton, C.B.E., who has been a member of the board for 32 years.

An interesting instalment of hospital history, which should be completed fitly to celebrate the Lynton Hospitals nearing jubilee, has appeared in the "Devon and Exeter Gazette." An early rule that a generation ago no one expected ever to see altered in any hospital, said that "there shall be no smoking."

The Rev. Hugh B. Chapman, chaplain of the Savoy, draws attention to "a small private hospital for gentlewomen run by a lady, without any expense to the patients," and will receive the names of any needing treatment who are unable to pay fees. Applications should be addressed to the Superintendent, Savoy Chantry, 7 Savoy-hill, W.C. 2.

The London University is postponing recognition of the courses at the Welsh School of Medicine until certain conditions are fulfilled. These include a larger number of beds at King Edward VII. Hospital, Cardiff; and the immediate effect may be the non-recognition of this year's work, which will lead students to transfer themselves to London medical schools.

Pandemonium is said to exist in the wards of the Lewisham Poor Law hospital, because the pressure on the beds has led to the overflow of mental patients into other wards. Two members after a visit said that the "din was sufficient to turn an ordinary brain." Dr. Nockolds, the medical superintendent, is understood often to have reported the conditions prevailing, but no other remedy is contemplated beyond such as securing accommodation at the Bermondsey Institution and at King's College Hospital, which the board has lately decided to seek.

The Leyton War Memorial Committee, which originally proposed to build a War Memorial Hospital for Leyton, has decided, we understand, that sufficient money cannot be raised. With £6,000 in hand, it proposes, subject to the approval of the Charity Commissioners, to endow beds at the Walthamstow Hospital for Leyton residents, and perhaps to spend the balance on a local memorial. In regard to the latter, free expert advice can be obtained from the Civic Arts Association.

The Duke of York visited Salford on November 23, to unveil the War Memorial tablet in the Salford Royal Hospital.

Dr. Robert Oswald Moon stood for Parliament in the Liberal interest for Wimbledon. He served with the French Legion in the Graeco-Turkish War, and was a Major in the R.A.M.C. on the Western and Salonican fronts in the Great War. He failed to be elected.

It was decided by a narrow majority not to appoint a special committee to inquire into the cause of the dismissals and resignations at the East Ham Borough Isolation Hospital.

The new sanatorium of the King Edward VII. Welsh National Memorial Association at Machynlleth is now nearly finished; and the transfer of the Pontsarn Sanatorium to the Association has been approved in principle by the Merthyr guardians.

The experiment adopted a year ago at the Birmingham Children's Hospital of admitting paying patients to private wards has been successful. The latest quarterly report states the committee's hope that the scheme eventually may largely solve the problem of the hospital's finances.

The opening of Devonshire House, Piccadilly, to the public at the modest charge of 1s. 3d., and the Hospitals Prize Competition (modelled on the Golden Ballot), for which tickets may be had for 5s. each, from the same address, should do much to raise the remainder of the £500,000 aimed at by the Hospitals of London Combined Appeal. At the moment of writing, £94,500 remains to be raised.

Andover Cottage Hospital has decided to adopt an "insurance" scheme like that in force at Winchester and Salisbury Hospitals, whereby regular contributors will be relieved of payment for maintenance when in hospital. The Andover audience had the good fortune to hear the details from Mr. Keyser, hon. treasurer of the Hants. County Hospital, who has done so much for the plan at Winchester. He is now addressing a series of meetings throughout the county.

The recent sale of books at Seaham Hall included one entitled "The Constitution and Rules of the Infirmary, Dispensary and House of Recovery for the Cure of Contagious Fever and Humane Society for the Towns of Bishopwearmouth, Sunderland, Monkwearmouth, and their immediate Vicinities," published by order of the committee, 1824. The dietary lays down, among other provisions: "Beer.—Every day. A child from four years to eight, 4 oz.; eight to 12, half a pint; from 12 to 16, 12 oz.; above 16, 1 pint." The rules enjoin that the patients on their discharge cured shall receive from the apothecary a form of thanks to Almighty God which they shall recite in their accustomed place of worship, and that they shall also return thanks to the governor who recommended them.

The difficulty of finding accommodation for child patients has led the Royal Devon and Exeter Hospital Committee to erect a Bungalow Ward, where the conditions for a return to health will be ideal. Sun-baths and fresh air, the beneficial effects of which are not sufficiently recognised by the majority of people, form a very important part of the treatment. The ward is situated in the Hospital Garden, and special wheeled cots have been provided, so that the little patients may be taken outside to enjoy the sunshine and bright, cheerful flowers. When they are well enough to get up they can play on the grass plot which has been sown for the purpose. The inside of the ward is also being made as attractive as possible, the walls are to be decorated with painted panels representing scenes from fairy stories, &c., with a stencilled dado and frieze. The Exeter School of Art has agreed to make itself responsible for this; the beaver boarding, &c., has been presented, and as the nucleus of the ward was an old army hut, it seems as if the maximum of usefulness will be obtained with the minimum of expense.

A War Memorial Hospital is to be built at Roseneath, Wrexham, at a cost of £70,000 for the Wrexham and East Denbighshire districts.

King's College Hospital has greatly benefitted by the harvest gifts of fruit, flowers, vegetables, bread and eggs, received from the churches and schools in the area of its work.

